# Synthesis and Characterization of Bone Binding Antibiotic-1 (BBA-1), a Novel Antimicrobial for Orthopedic Applications

**DOI:** 10.3390/molecules26061541

**Published:** 2021-03-11

**Authors:** Sumedh Kamble, Peter Valtchev, Aiken Dao, Théophile Pelras, Michael J Rogers, Paul B. Savage, Fariba Dehghani, Aaron Schindeler

**Affiliations:** 1 Bioengineering and Molecular Medicine Laboratory, Kids Research at The Children’s Hospital at Westmead, Westmead, NSW 2145, Australia; sumedh.kamble@sydney.edu.au (S.K.); aiken.dao@sydney.edu.au (A.D.); 2 Discipline of Child and Adolescent Medicine, Sydney Medical School, University of Sydney, Sydney, NSW 2050, Australia; 3 School of Chemical and Biomolecular Engineering, University of Sydney, Camperdown, NSW 2008, Australia; peter.valtchev@sydney.edu.au (P.V.); fariba.dehghani@sydney.edu.au (F.D.); 4 Key Centre for Polymers and Colloids, School of Chemistry and Sydney Nano, University of Sydney, Camperdown, NSW 2006, Australia; theophile.pelras@sydney.edu.au; 5 Garvan Institute of Medical Research, St. Vincent’s Clinical School, Faculty of Medicine, University of New South Wales, Darlinghurst, NSW 2010, Australia; m.rogers@garvan.org.au; 6 Department of Chemistry and Biochemistry, Brigham Young University, Provo, UT 84601, USA; pbsavage@chem.byu.edu

**Keywords:** BBA-1, bone infection, osteomyelitis, CSA-90, alendronate, antimicrobial

## Abstract

Osteomyelitis and orthopedic infections are major clinical problems, limited by a lack of antibiotics specialized for such applications. In this paper, we describe the design and synthesis of a novel bone-binding antibiotic (BBA-1) and its subsequent structural and functional characterization. The synthesis of BBA-1 was the result of a two-step chemical conjugation of cationic selective antimicrobial-90 (CSA-90) and the bisphosphonate alendronate (ALN) via a heterobifunctional linker. This was analytically confirmed by HPLC, FT-IR, MS and NMR spectroscopy. BBA-1 showed rapid binding and high affinity to bone mineral in an in vitro hydroxyapatite binding assay. Kirby—Baur assays confirmed that BBA-1 shows a potent antibacterial activity against *Staphylococcus aureus* and methicillin-resistant *S. aureus* comparable to CSA-90. Differentiation of cultured osteoblasts in media supplemented with BBA-1 led to increased alkaline phosphatase expression, which is consistent with the pro-osteogenic activity of CSA-90. Bisphosphonates, such as ALN, are inhibitors of protein prenylation, however, the amine conjugation of ALN to CSA-90 disrupted this activity in an in vitro protein prenylation assay. Overall, these findings support the antimicrobial, bone-binding, and pro-osteogenic activities of BBA-1. The compound and related agents have the potential to ensure lasting activity against osteomyelitis after systemic delivery.

## 1. Introduction

Management of orthopedic infections and osteomyelitis are challenging due to the high morbidity of such conditions, the limited antibiotic options, and an ever-increasing prevalence of antibiotic resistant pathogens [1,2]. Traumatic open fractures and joint replacement surgeries are situations that present with the highest risk of infection in an orthopedic setting [3,4]. Despite improvements in orthopedic surgical techniques, the rate of infection following arthroplasty procedures remains high and results in a disproportionate healthcare burden [5,6]. While systemic antibiotics can resolve acute cases of osteomyelitis, chronic osteomyelitis can remain recalcitrant even with high and sustained antibiotic doses [7]. Moreover, antibiotic efficacy can be further limited by bacterial biofilms, vascular insufficiency, poor antibiotic penetration into the bone, and the complexity of the bone microenvironment [8]. Due to the serious clinical consequences of inadequate treatment, there is a drive to develop novel therapeutics to treat bone infection. We speculated that the bone-targeting of antimicrobials could achieve sustained bone activity, while limiting systemic exposure.

Cationic selective antimicrobials (CSAs) are synthetic antimicrobial agents, structurally based on cholic acid. Due to their strong amphiphilic nature and cationic characteristics, CSAs often surpass the antibacterial activities of endogenous antimicrobial peptides, such as cathelicidin LL-37 [9,10]. CSAs also have improved stability and reduced in vivo toxicity [9]. CSA-90 was identified as a candidate orthopedic antimicrobial based on its potent and broad-spectrum antibacterial action against both Gram-positive and Gram-negative bacteria, including their resistant strains [11,12]. Prior studies have shown local dosing to be prophylactic for *S. aureus* infection in preclinical rat fracture models [11,12]. Additionally, the pro-osteogenic effects of CSA-90 could be beneficial for improving bone repair and/or orthopedic implant osseointegration [11].

Bisphosphonates (BPs) are structural analogues of inorganic pyrophosphate that have a high and selective affinity towards hydroxyapatite (HA; bone mineral). While BPs are clinically used for the treatment of metabolic bone conditions and prevention of skeletal-related tumor events [13], it has been speculated that BP conjugation could be used as a drug targeting moiety [14]. In terms of HA-binding capacity, second generation amino-BPs (N-BPs), such as alendronate (ALN), have stronger a bone-binding affinity than non-amino BPs [15,16]. The utility of BPs as bone targeting agents is further enhanced by their tendency to concentrate at sites of high bone turnover, which is the case for osteolytic bone infections [14,17]. A variety of factors can influence the functionality of a conjugated BP in bone targeting, including the choice of BP moiety, the nature of the chemical reaction for conjugation, the properties of any linker(s), and the pharmacological stability of the final conjugate [17].

In this report, we describe the synthesis and characterization of a novel ALN to CSA-90 conjugate termed “bone-binding antimicrobial-1” (BBA-1). This involved a two-step reaction, incorporating a heterobifunctional poly(ethylene glycol) (PEG) linker. Evidence suggests that PEG linkers are able to produce stable drug conjugates, limit nonspecific uptake, and facilitate tissue targeting by enhanced permeability and retention [18]. BBA-1 was hypothesized to retain an antimicrobial activity comparable to its parent compound CSA-90, yet acquire a strong affinity for bone mineral. Following physicochemical characterization of BBA-1, in vitro assays for antimicrobial activity and bone mineral affinity were performed. Additionally, the osteogenic activity of BBA-1 was tested in an osteoblast culture model [12], and its effect on protein prenylation (that underlies the ability of N-BPs to inhibit bone resorption) was also examined [19].

## 2. Results

### 2.1. Identification of Synthesized BBA-1 Using HPLC

**Figure 1:**
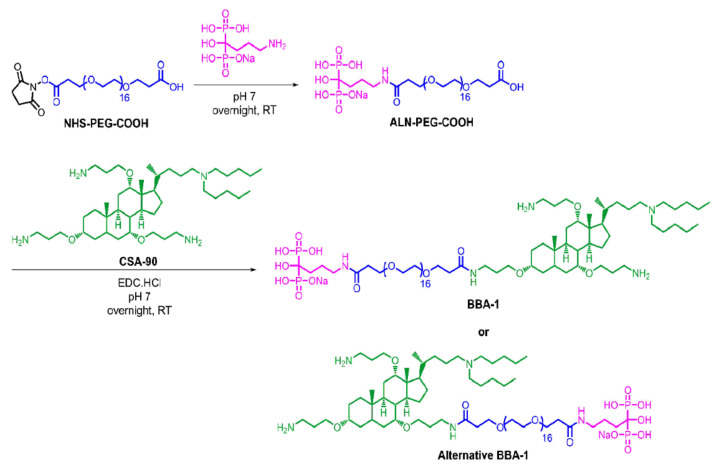
Synthesis of bone-binding antibiotic (BBA-1) by conjugating alendronate (ALN) and cationic selective antimicrobial-90 (CSA-90) using the NHS-PEG-COOH linker.

A schematic representing the overall synthesis procedure of BBA-1 is depicted in Figure 1. The conjugation of ALN to the N-hydroxy succinimide (NHS) termini and CSA-90 to the carboxylic termini of the NHS-PEG-COOH linker were monitored and identified by HPLC.

**Figure 2:**
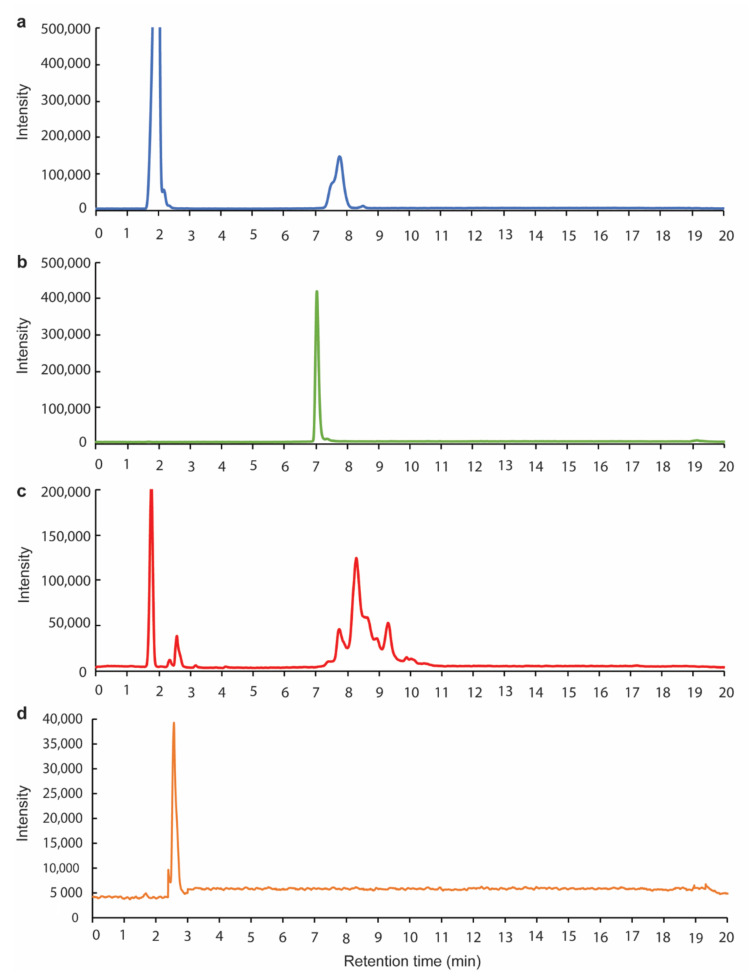
HPLC chromatograms of (**a**) NHS-PEG-COOH linker, (**b**) CSA-90, (**c**) synthesized and purified BBA-1 and (**d**) 1-ethyl-3-(3-dimethylaminopropyl) carbodiimide hydrochloride (EDC.HCl).

In addition to the appearance of a peak corresponding to the NHS-PEG-COOH linker eluting at 7.5 min, the reaction product eluted between 8–10 min with distinct peaks at 8.2 min and 9.3 min (Figure 2c). These separate peaks were attributed to the alternate conjugation of ALN-PEG-COOH to the two primary amine groups of CSA-90. A peak at 2.5 min was the result of residual 1-ethyl-3-(3-dimethylaminopropyl) carbodiimide hydrochloride (EDC.HCl) from the conjugation reaction (Figure 2c,d). These peak identities are consistent with the corresponding elution profiles of pure NHS-PEG-COOH linker and CSA-90 (Figure 2a,b). The absence of a 7 min CSA-90 peak in Figure 2c indicates that the conjugation of CSA-90 to the linker was highly efficient. The peak at ~2 min (Figure 2a,c) was attributed to an impurity in the NHS-PEG-COOH linker.

### 2.2. Characterization of BBA-1 Using Infra-Red Spectroscopy

**Figure 3:**
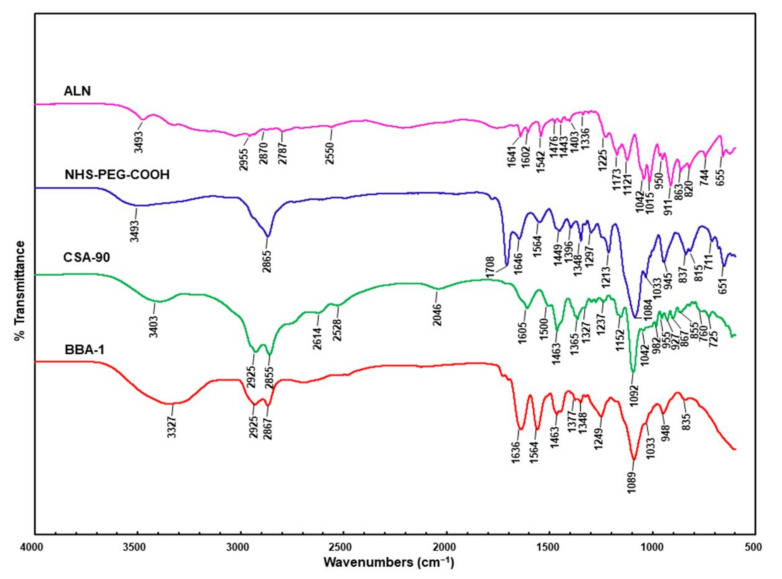
FT-IR spectra of ALN, NHS-PEG-COOH, CSA-90 and BBA-1. Formation of BBA-1 is indicated by the presence of a typical carbonyl group at 1636 cm^−1^ due to the stretching vibration of the amide formed after alendronate and CSA-90 conjugation.

The FT-IR spectroscopy was used to further characterize the BBA-1 synthesis product. In all the spectra, (Figure 3)) peaks at 1550–1560 cm^−1^ and above 3300 cm^−1^ correspond to N-H bending and stretching vibrations, respectively. The presence of peaks at 2867 and 2925 cm^−1^ in the spectrum of BBA-1 (Figure 3) correspond to the C-H stretching modes of ethyl and alkyl groups in CSA-90 that are retained in the synthesized BBA-1. The conjugation of CSA-90 and ALN via the NHS-PEG-COOH linker was confirmed by the appearance of stretching vibrations of the carbonyl group of the amide bond at 1636 cm^−1^ (Figure 3 BBA-1).

### 2.3. Characterization of BBA-1 by NMR Spectroscopy

**Figure 4:**
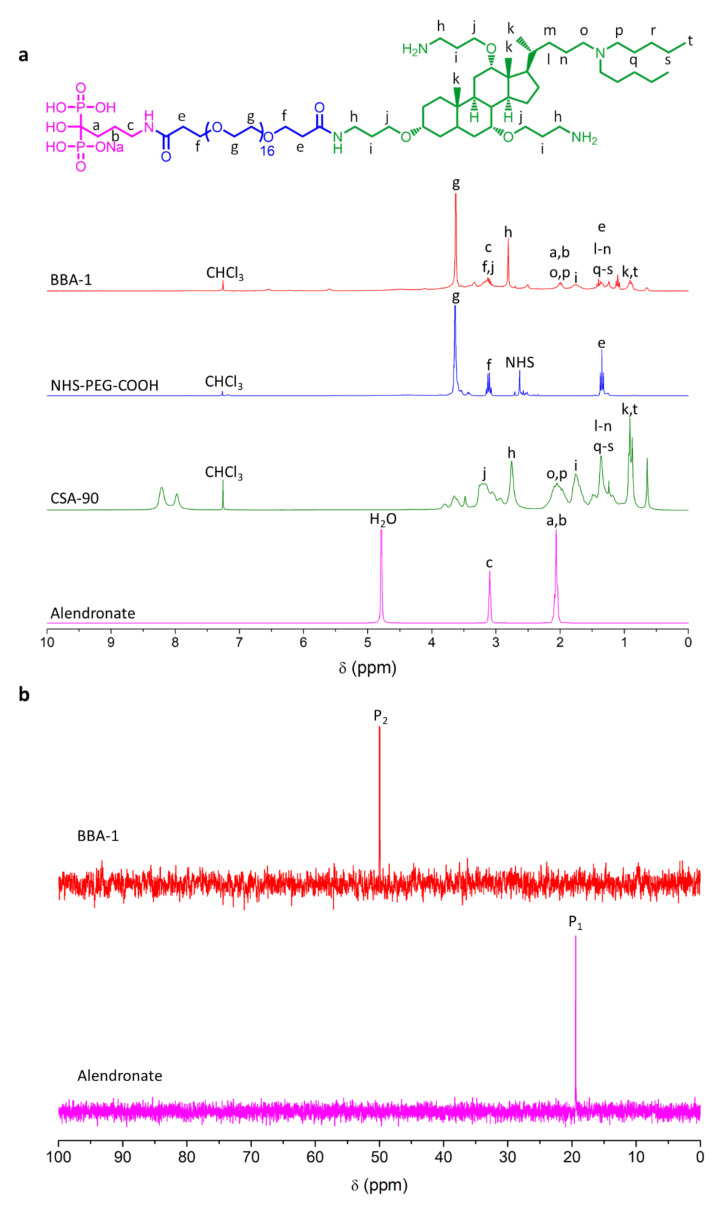
^1^H-NMR and ^31^P-NMR characterization. (**a**) ^1^H-NMR spectra of BBA-1, NHS-PEG-COOH linker, CSA-90 and alendronate. (**b**) ^31^P-NMR of BBA-1 and ALN.

Proton and phosphorus NMR spectroscopy were employed to confirm the detailed structure of BBA-1 (Figure 4a,b). The ^1^H-NMR spectrum recorded for synthesized BBA-1 exhibited proton signals characteristic of the methylene protons from the ethylene oxide linker (g, 3.64 ppm). The disappearance of the triplet characteristic to CH_2_-CH_2_ from the NHS heterocycle from the NHS-PEG-COOH linker (NHS, 2.64 ppm) confirmed ALN conjugation to NHS termini of the linker. Conjugation of CSA-90 to the carboxylic terminus of ALN-PEG-COOH was confirmed by the appearance of characteristic peaks of CSA-90 at 0.93 ppm (k and t, -CH_3_), 1.25 ppm (l, m, n, q, r and s, -CH_2_), 1.76 ppm (i, -CH_2_) and 2.82 ppm (h, -CH_2_). BBA-1 ^1^H-NMR (CHCl3): delta 3.64 ppm (PEG CH_2_); 3.21 ppm (CSA-90 -CH_2_-O-); 2.76 ppm (CSA-90 H_2_N-CH_2_-); 2.05 ppm (CSA-90 -CH_2_-N-); 1.76 ppm (CSA-90 -CH_2_-); 1.36 ppm (CSA-90 -CH- and -CH_2_-) and 0.91 ppm (CSA-90 -CH_3_). The peaks at 7.9 and 8.2 ppm in the CSA-90 spectrum were attributed to minor impurities which were removed during the purification of the conjugate molecule. The characteristic signals of alendronate (a,b, 2.05 ppm and c, 3.10 ppm) are superimposed by the many proton signals from the conjugated CSA-90, therefore, conjugation was further verified by ^31^P-NMR. Phosphorus, present within ALN (P_1_, 19.42 ppm), shifted its peak to a higher value (P_2_, 50.06 ppm) in the BBA-1 spectra (Figure 4b). This unequivocally demonstrates the successful incorporation of ALN into the conjugate molecule.

### 2.4. Confirmation of Synthesized BBA-1 by Mass Spectrometry (MS)

**Figure 5:**
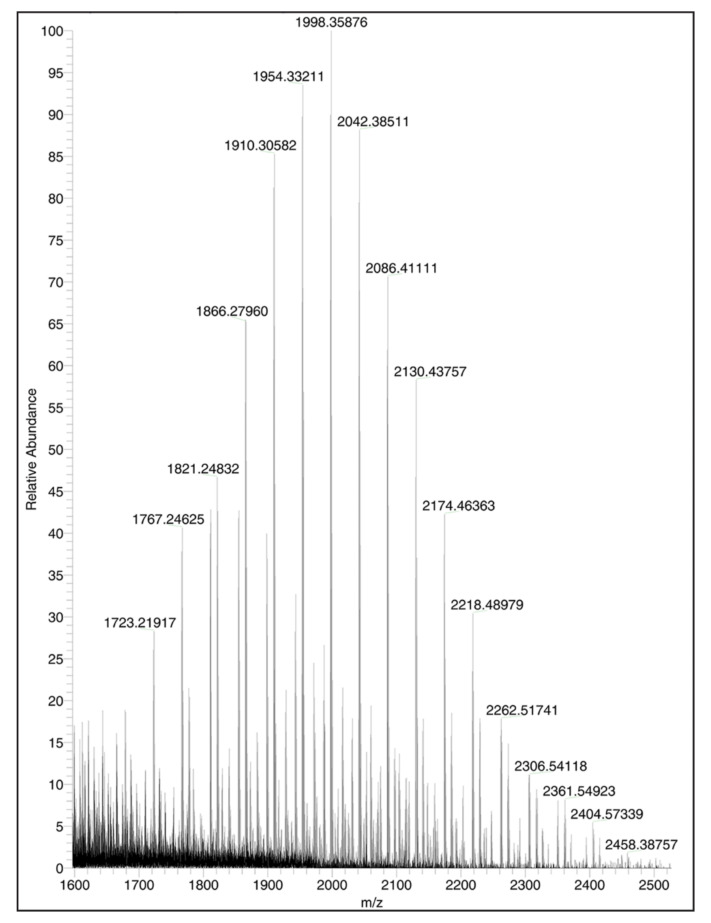
Mass spectrum of BBA-1. Synthesis of BBA-1 after CSA-90 and ALN conjugation is evident by the presence of distinctive ions corresponding to total mass of CSA-90, NHS-PEG-COOH linker and alendronate conjugation (BBA-1) spaced by *m/z* of ~44 arising from the polydispersity of the PEG linker.

MS characterization of CSA-90, ALN and BBA-1 was performed to further confirm the synthesis and structure of BBA-1 (Figure 5, MS of CSA-90 and ALN Supplementary Materials). The mass-spectrum of the product demonstrated a prominent group of peaks with a median *m/z* of 1998, corresponding to BBA-1 singly conjugated species. No doubly conjugated species were observed, nor were peaks with *m/z* corresponding to triply conjugated product (checked with extended mass calibration of the orbitrap, data not shown). The peaks corresponding to BBA-1 were spaced by *m/z* of 44 (-CH_2_-CH_2_- monomer unit), which is attributed to the polydispersity of the PEG linker.

### 2.5. BBA-1 Binding to Hydroxyapatite

Since HA is the major component of bone mineral, HA microparticles were used to assess the bone binding affinity of BBA-1 in vitro. An assay was performed based on the previously published method, with some modifications [20]. BBA-1 and CSA-90 were incubated with and without HA in Milli-Q water and subsequently measured in the aqueous phase of the suspension using HPLC. It was hypothesized that BBA-1 would be proportionately reduced in concentration due to its binding/adsorption to the HA surface, but CSA-90 would be unaffected.

**Figure 6:**
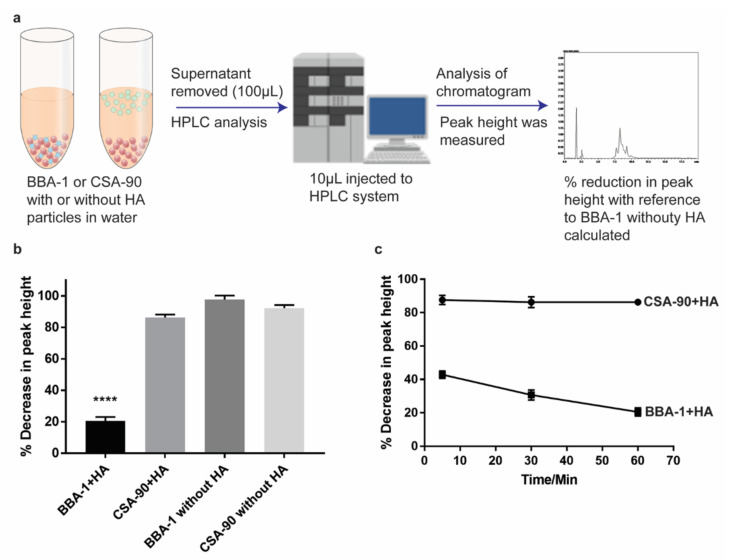
Hydroxyapatite (HA) binding affinity of BBA-1 and parent compound CSA-90. (**a**) Schematic of the experimental procedure. (**b**) HA affinity of BBA-1 and CSA-90 after 60 min of incubation in HA suspension. (**c**) Binding kinetics for BBA-1 and CSA-90 on HA over 60 min. **** Significantly different (*p* < 0.001, Tukey’s multiple comparison test, one-way ANOVA, *n* = 3 independent measurements).

The amount of BBA-1 binding was determined by measuring the relative peak height of the initial HA-free BBA-1 solution used as a control versus the peak height obtained from the aqueous phase after HA addition. After 60 min of incubation, the peak height of BBA-1 in the aqueous phase was reduced by ~80% (Figure 6b). In contrast, CSA-90 with HA showed no specific bone mineral binding. The difference between BBA-1 and CSA-90 was highly significant (*p* < 0.001). A subsequent time series showed that binding was rapid, with a ~40% decrease in peak height within the first 5 min of incubation with HA (Figure 6c). These findings explicitly demonstrate that BBA-1 has considerable bone mineral affinity.

### 2.6. BBA-1 Has Antimicrobial Activity against Staphylococcus aureus

**Figure 7:**
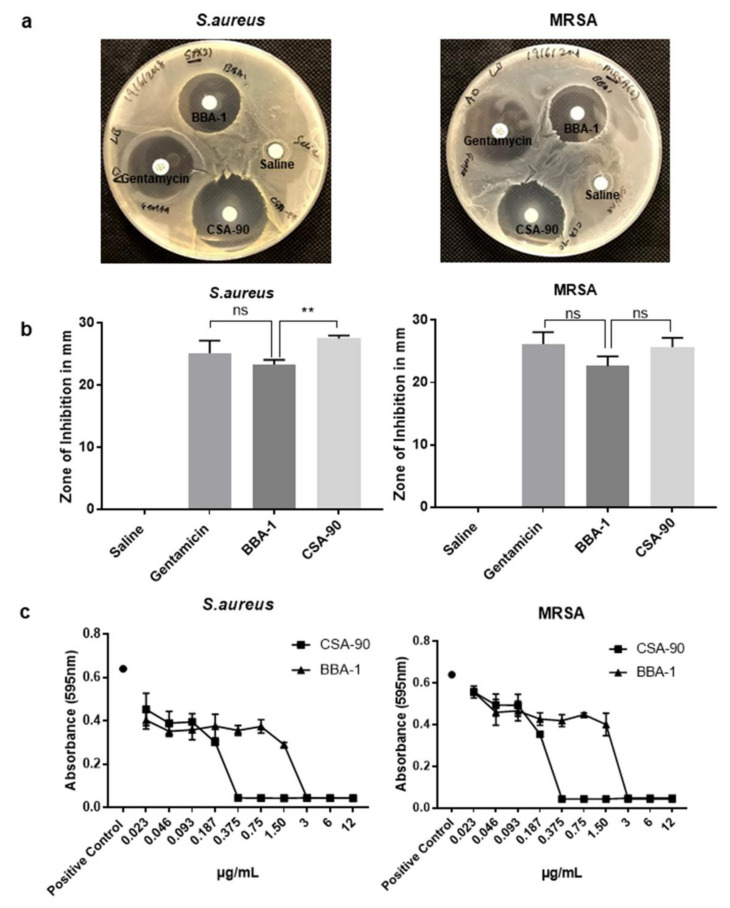
Antimicrobial susceptibility test and determination of minimum inhibitory concentration against *S. aureus* and methicillin-resistant *S. aureus* (MRSA). (**a**,**b**) Bacterial growth inhibition by Kirby-Bauer disc diffusion assay against *S. aureus* and MRSA (** *p* < 0.01, ns—not significant). (**c**) Determination of minimum inhibitory concentration (MIC) and minimum bactericidal concentration (MBC) value of BBA-1 and CSA-90 against *S. aureus* and MRSA (all measurements performed in triplicate).

To assess the antibacterial effect of synthesized BBA-1, a modified Kirby—Bauer disc diffusion assay was performed as per the previously published method [11]. BBA-1 treated discs significantly inhibited bacterial growth in vitro in a modified Kirby—Bauer disc diffusion assay against *Staphylococcus aureus* (*S. aureus*) and methicillin-resistant *S. aureus* (MRSA) across all experiments (Figure 7a,b). The antibacterial activity of BBA-1 at an equimolar concentration of CSA-90 and gentamicin was found to be comparable against both species of bacteria. Discs containing 200 µg gentamicin were used as a positive control for this experiment; gentamicin as one of the few local antibiotics suitable for local treatment of orthopedic infections. The minimum inhibitory concentration (MIC) and minimum bactericidal concentration (MBC) determined by standardized broth microdilution method against both *S. aureus* and MRSA, for BBA-1 were 1.5 and 3 µg/mL, respectively (Figure 7c). In contrast, the MIC and MBC values for CSA-90 were 0.187 and 0.375 µg/mL, respectively (Figure 7c), for both bacterial strains.

### 2.7. BBA-1 Promotes Osteogenic Differentiation in Cultured Osteoblast

**Figure 8:**
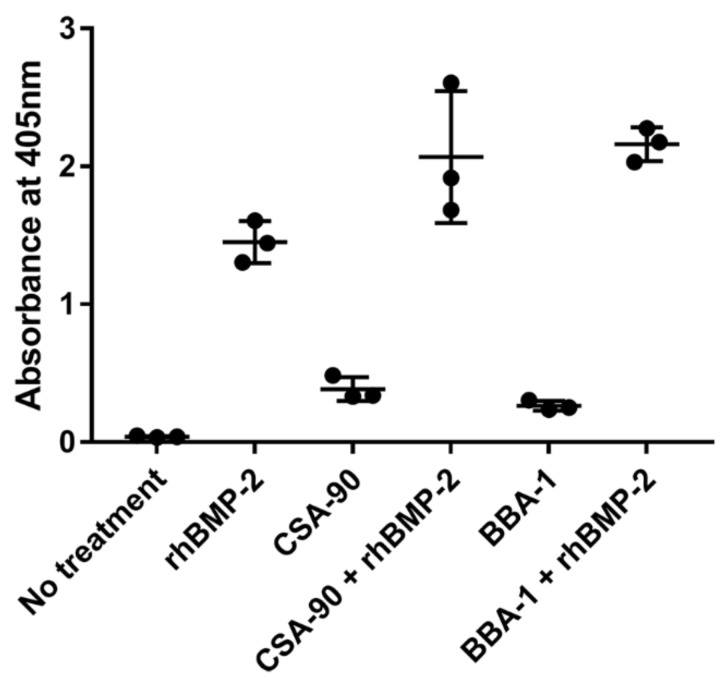
Quantitative spectroscopic alkaline phosphatase assay. BBA-1 showed a similar effect on alkaline phosphatase activity alone and in combination with rhBMP-2 as CSA-90 in MC3T3-E1 pre-osteoblast cells.

Alkaline phosphatase is a major regulator of bone mineralization. Its increased expression is an indicator of osteogenic differentiation, where it can be quantified using a colorimetric para-nitrophenyl phosphate (pNPP) assay at λmax 405 nm [21]. CSA-90 has been shown to have intrinsic osteogenic activity and potentiates rhBMP-2 induced bone cell differentiation in culture [12]. To test whether BBA-1 retained any of the pro-osteogenic action of CSA-90, MC3T3-E1 cells were differentiated in osteogenic media with either 5 µM CSA-90 or BBA-1, with or without 50 ng/mL rhBMP-2. Consistent with prior data [12], CSA-90 and rhBMP-2 increased alkaline phosphatase expression as a marker of osteogenic differentiation (Figure 8). BBA-1 showed similar osteogenic potential to CSA-90 both alone and in combination with rhBMP-2. Thus, CSA-90 retains its osteogenic action, even when conjugated to ALN via the PEG linker in BBA-1.

### 2.8. BBA-1 Does Not Impair Protein Prenylation

**Figure 9:**
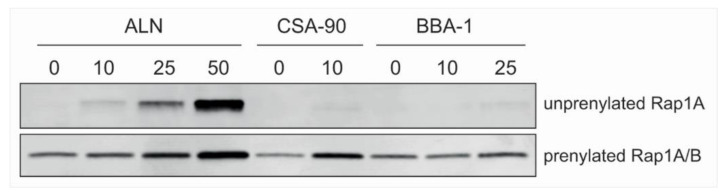
Effect of BBA-1 on protein prenylation in J774.2 macrophage cells. ALN treatment inhibited Rap1A prenylation in J774.2 cells at 10 µM or above but CSA-90 and BBA-1 had negligible effects. Prenylated Rap1A/B was used as a loading control.

ALN is a potent inhibitor of protein prenylation [22], an effect that can be quantified by detecting the accumulation of unprenylated Rap1A by Western blotting [19]. Concentrations ≥10 µM ALN dose-dependently inhibited the prenylation of Rap1A GTPase in J774.2 cells (Figure 9). Conversely, treatment with an equimolar concentration of CSA-90 (up to 10 µM) or up to 25 µM BBA-1 had a negligible effect on Rap1A prenylation. Testing of higher concentrations of CSA-90 (≥25 µM) or BBA-1 (≥50 µM) was not feasible due to cell toxicity. These data indicate that ALN conjugation to CSA-90 via the primary amine group of ALN blocks the ability to inhibit protein prenylation. Moreover, any marginal effects of BBA-1 on prenylation were speculated to be due to residual ALN present in the BBA-1 preparation that was not fully removed by the purification process.

## 3. Discussion

This study describes the design, synthesis and characterization of BBA-1 as a novel bone targeting antimicrobial agent for treatment of orthopedic infections. The broad-spectrum potent antibacterial activity and pro-osteogenic effects of CSA-90 are particularly appealing for use in orthopedic settings for the prevention and treatment of osteomyelitis. Conjugation of CSA-90 to ALN via a PEG-based heterobifunctional linker is a promising approach for achieving sustained and targeted delivery to bone. The sites used for conjugation in both compounds were specifically chosen in order to retain the bone binding and antimicrobial properties, respectively. The primary amine in ALN was the preferred conjugation site, in order to retain the P-C-P backbone pharmacophore for its avid bone affinity and binding to HA [15]. Conversely, maintaining the structural arrangements at the C24 position in CSA-90 was key for preserving its antimicrobial activity, as this residue has been linked to bacterial membrane depolarization and disruption [23].

Ultimately, a heterobifunctional linker was adopted to link CSA-90 and ALN. Several prior studies have reported the use of homobifunctional linkers for -NH2 to -NH2 conjugations [24,25], but such types of reactions have intrinsic limitations. The yield and purity of the final conjugate can be poor and it requires extensive subsequent purification. Indeed, our initial trials using bis(sulfosuccin-imidyl)suberate, a classic homobifunctional linker for -NH2 to -NH2 conjugation, showed limited conjugation efficiency (data not shown). In contrast, using an NHS-PEG-COOH heterobifunctional linker, we observed maximum conjugation efficiency for both ALN and CSA-90. While our study did not directly measure the extent of ALN conjugation to the NHS group or CSA-90 to the COOH termini of the NHS-PEG-COOH linker, the complete disappearance of CH2 signals from the NHS termini in the 1H-NMR spectra of BBA-1 suggests a maximal conjugation efficiency. Furthermore, MS analysis demonstrated singly conjugated species spaced by *m/z* of 44 (-CH2-CH2- monomer unit), indicating that the conjugation of CSA-90 occurred at the least sterically hindered terminal amino group at the C3 carbon atom from the cyclopentano perhydro phenanthrene ring. Nevertheless, the terminal alkyl amine at the C7 position also remains a possible conjugation site compared to the third terminal amino group conjugated to C12, which is sterically hindered by the adjacent bulky tertiary propyl amine at C17.

BBA-1 possesses a robust antibacterial activity, as shown by the modified Kirby—Bauer disc diffusion assays against *S. aureus* and MRSA, which are frequently isolated bacteria from most clinical cases of orthopedic infection [26,27]. Data from MIC and MBC testing against *S. aureus* and MRSA confirms that BBA-1, similarly to CSA-90, has bactericidal activity. The covalent linkage of CSA-90 to ALN via the PEG linker had minimal impact on the antibacterial activity. Although a higher absolute concentration of BBA-1 was required to reach the MIC due to its significantly increased molecular weight, its antibacterial activity was comparable to the parent compound CSA-90 when used at an equimolar concentration. BBA-1 also showed a comparable antibacterial activity to gentamicin, another antimicrobial used in the treatment of *S. aureus* and MRSA bone infection. Moreover, CSA-90 has antimicrobial activity against a range of pathogens via a similar mechanism, so it would not be presumptuous to extrapolate maintenance of generalized antibiotic action.

There have been prior attempts to target antibiotics to bone using chemical conjugation, although these have used a cleavable approach to attempt to release the drug at the site of action [28,29]. The rationale for this strategy was that fluoroquinolone antimicrobials used in the aforementioned studies lose antibacterial activity upon conjugation. In the first of these studies, ciprofloxacin was conjugated to a bisphosphonate using a cleavable carbamate linker to target osteomyelitis biofilms [28]. The conjugated compound had a significant increase in MIC, reflective of a reduced antimicrobial activity. However, a single dose of 10 mg/kg was found to reduce bacterial load by 99% in an animal model of periprosthetic infection. In contrast, the conjugation of CSA-90 to ALN did not considerably reduce its MIC50 (1.5 µg/mL) or MBC (3 µg/mL). BBA-1 was not designed to release CSA-90 after binding but rather have long term action; several studies have indicated that non-cleavable drug conjugates outperform their cleavable counterparts due to improved plasma stability and improved target specificity [30,31].

As part of the characterization of BBA-1 it was critical to analyze the multiple functional activities of CSA-90 and ALN. These included the antimicrobial and pro-osteogenic activities of CSA-90 and the bone-binding and protein prenylation-inhibiting activities of ALN. Having demonstrated antibacterial activity of BBA-1, we set out to test the pro-osteogenic activity of BBA-1 in MC3T3-E1 pre-osteoblasts. In this culture system, CSA-90 and BBA-1 were found to increase alkaline phosphatase expression alone under conditions of bone cell differentiation and showed additional effects upon the addition of rhBMP-2. The capacity for CSA-90 to increase bone formation in vivo has been shown in rats [12], however, this needs to be further validated in implant-associated infections, since implants are highly vulnerable to biofilm formation and loss of fixation [32]. BBA-1 has no specific affinity for metal surfaces, but it may have additional applications in the context of ceramic implants that feature calcium phosphate or analogues that BBA-1 would bind to.

An in vitro HA-binding assay was used to show that >80% of BBA-1 bind to HA microparticles within 1 h. A limitation of this methodology is that it indirectly measures the reduction in BBA-1 in the aqueous phase rather than HA-bound BBA-1. Nevertheless, our findings remain consistent with a prior report stating that ~80–90% of a bisphosphonate conjugate bound to the solid phase within 1 h of incubation [33]. Previous studies have suggested that modifications to the primary amine group of ALN led to loss of its anti-resorptive activity while the bone-targeting potential remains unaffected [15]. The anti-resorptive potency of N-BPs, such as ALN, is determined by their ability to inhibit farnesyl diphosphate synthase and thereby block protein prenylation [22]. We, therefore, examined whether BBA-1 affected protein prenylation using a Western blot approach to detect unprenylated Rap1A. Whereas ALN treatment led to a clear increase in unprenylated Rap1A protein in macrophages, BBA-1 and CSA-90 did not. Notably, higher concentrations of CSA-90 and BBA-1 were limited by the cytotoxic effects of these compounds on cultured J774.2 cells, which have been previously described for CSAs [34]. These cytotoxic effects of CSAs can limit their systemic dosing, which will be a consideration for the future delivery of BBA-1. At higher systemic concentrations, CSAs have been shown to affect red blood cells (RBCs) and cause hemolysis [9,35]. Nevertheless, poloxamer carriers or sustained delivery systems are able to limit the toxicity of CSAs, making systemic BBA-1 dosing a viable therapeutic strategy.

This report describes the methods for BBA-1 drug synthesis and structural and in vitro functional testing. Our study describes only a single compound, but by varying the bisphosphonate, linker, or CSA it is possible that a range of BBA compounds could be produced. These could have more favorable antimicrobial, bone binding or pharmacokinetic/pharmacodynamic profiles. The current studies have not yet addressed the pharmacology of binding or antimicrobial functionality in preclinical models or the efficacy in treating models of osteomyelitis. These topics have been identified as the goals of future research studies.

## 4. Materials and Methods

### 4.1. Materials

CSA-90 (molecular weight 851 g/mol) was produced by Dr. Paul Savage’s laboratory at Brigham Young University (Provo, UT, USA). Alendronate sodium trihydrate was sourced from Alcon Biosciences India. NHS-PEG-COOH linker (molecular weight 1000 g/mol, *n* = 16) was purchased from NANOCS, New York, NY, USA. Ethanol (≥99.5%), acetonitrile (≥99.9%), 1-ethyl-3-(3-dimethylaminopropyl) carbodiimide hydrochloride (EDC.HCl), formic acid (FA, 98–100%) were purchased from Sigma-Aldrich, Castle Hill, NSW, Australia. Purified deionized water was prepared using the Milli-Q system. Hydroxyapatite (HA, particles size range 50–150 µm) was purchased from Berkeley Advanced Biomaterials, Berkeley, CA, USA. Recombinant human bone morphogenetic protein-2 (rhBMP-2, INFUSE Bone Graft Kit) was purchased from Medtronic Australasia (North Ryde, NSW, Australia).

### 4.2. Synthesis of ALN-PEG-COOH

Initially, a solution of alendronate sodium trihydrate (10.5 mg, 32.3 µmol into 1 mL Milli-Q water) was added dropwise into the solution of NHS-PEG-COOH (33 mg, 33.0 µmol in 1 mL Milli-Q water) under continuous stirring. The pH of the reaction mixture was monitored (pH 4.5 was observed) and adjusted to 7 using dilute NaOH solution. After an overnight reaction at room temperature (under continuous stirring using magnetic bar), the unconjugated ALN was removed by precipitation in 3.5 mL absolute ethanol. Precipitated ALN was removed by filtration (0.45 µm nylon filter). The resultant solution of ALN-PEG-COOH was then used in the next step for CSA-90 conjugation.

### 4.3. Conjugation of CSA-90 to the Carboxylic Terminal of ALN-PEG-COOH

CSA-90 was conjugated to the carboxyl terminal of ALN-PEG-COOH using water soluble carbodiimide catalyst. Briefly, EDC.HCl (30 mg, 157 µmol) and CSA-90 (25.5 mg, 36.2 µmol) were added to the solution of ALN-PEG-COOH. The reaction mixture was then stirred for 24 h at room temperature. The resultant ALN-PEG-CSA-90 conjugate (BBA-1) was dialyzed for 24 h using a Mini Dialysis Kit (1 kDa cut-off) obtained from GE Healthcare Bio-sciences, Princeton, NJ, USA (catalogue number 80-6483-94). The goal was to remove any unconjugated CSA-90 and excess of EDC.HCl or any intermediate product formed during the reaction. The purified product was subsequently freeze-dried for use in further studies.

### 4.4. Identification and Characterization of ALN-PEG-CSA-90 Conjugate (BBA-1)

The ^1^H-NMR spectra (BBA-1, NHS-PEG-COOH and CSA-90 in CDCl_3_, and ALN in D_2_O) were recorded on a 400 MHz INOVA spectrometer (Varian, Palo Alto, CA, USA) using tetra-methyl silane (TMS) as an internal standard. Deuterated solvents (CDCl_3_ and D_2_O) were purchased from Novachem Australia (Heidelberg West, VIC, Australia). The mass spectrometry (MS) characterization was performed on an Orbitrap Velos Pro hybrid MS spectrometer (Thermo Fisher Scientific, Waltham, MA, USA). The sample was dissolved in methanol and syringe infused into an electrospray ionization source connected to the mass spectrometer running in positive ion mode and scans over a minute were summed together. For detailed analysis and improvements in the sensitivity in the area of interest, the mass range was modified to 1500–2700 *m/z*. The synthesized BBA-1 was characterized by Fourier-transform infra-red (FT-IR) spectroscopy (Shimadzu 8400s FT-IR spectrometer Kyoto, Kyoto, Japan). For each sample, 45 scans were recorded as % transmittance at resolution of 4 cm^−1^ with a scanning span of 600–4000 nm. Reverse phase high-performance liquid chromatography (HPLC) employed for identification and characterization of BBA-1 was performed using a Shimadzu 20A series high-pressure mixing HPLC system equipped with an evaporative light scattering detector (ELSD, Varian 385-LC, Agilent Technologies, Santa Clara, California, USA). BBA-1 was dissolved in milli-Q water, and 10 µL of sample was injected into the HPLC system. BBA-1 was separated on a C18 column (Phenomenex^®^, Synergi^™^, 250 × 4.6 mm) maintained at 30 °C using mobile phase A (water + 0.1% FA) and B (acetonitrile + 0.1% FA) at a flow rate of 1.5 mL/min and a gradient elution (0–2 min B:5%; 2–10 min B:5–85%; 10–15 min B:85%, 15–17 min B:5%, 17–20 min B: 5%).

### 4.5. In Vitro Bone Mineral Affinity Assay

The affinity of BBA-1 and the parent compound CSA-90 for bone mineral were tested in vitro based on the previously published protocols [20]. Briefly, BBA-1 (2.42 mg) was weighed and dissolved in 4 mL Milli-Q water. Separately, HA microparticles (20 mg) were weighed and suspended in 2 mL BBA-1 solution. The BBA-1 + HA suspension was incubated at room temperature. After predetermined time intervals, 100 µL of supernatant from the HA + BBA-1 suspension and BBA-1 alone was withdrawn and 10 µL of supernatant was injected into the HPLC system. The peak heights of BBA-1 or CSA-90 were used for relative quantitation. Similarly, bone binding ability of parent CSA-90 was also tested by incubating it with and without HA at similar concentrations. The binding percentage, corresponding to the percentage reduction in peak height, was calculated based on the following formula:

[(Peak Height _without HA_ − Peak Height _with HA_)/(Peak Height _without HA_)] × 100% (1)

### 4.6. Antibacterial Activity of BBA-1

To compare and test the bactericidal activity of synthesized BBA-1, modified Kirby—Bauer disc diffusion assay was performed using lysogeny broth (LB) agar plates. Whatman filter paper discs N^Ø^1 (6 mm) containing BBA-1 and CSA-90 equimolar to standard antibiotic gentamicin (200 µg/disc) were prepared. Briefly, 0.5 mL of bacteria—either *S. aureus* (ATCC (American Type Culture Collection)-12600) and methicillin resistant *S. aureus* (MRSA clinical isolate, obtained from Prof David Isaacs, The Children’s Hospital at Westmead, NSW, Australia) [11]—diluted to an OD_600_ of 0.2 (i.e., ~1.6 × 10^8^ cfu/mL) were spread evenly on LB agar plates. Antibiotic discs were then placed on the inoculated LB agar plates and the zone of bacterial growth inhibition due to each antibiotic disc was measured in mm after overnight incubation at 37 °C.

### 4.7. Determination of Minimum Inhibitory Concentration (MIC) and Minimum Bactericidal Concentration (MBC)

These metrics were determined for BBA-1 by the standardized broth microdilution method. Briefly, 1 × 10^5^ CFU/mL of *S. aureus* and MRSA inoculum was incubated with serial concentrations of BBA-1 and CSA-90. The optical density/absorbance was recorded in triplicate at 595 nm after 24 h of incubation at 37 °C.

### 4.8. Cell Culture and Osteogenesis Assays

The pro-osteogenic activity of BBA-1was tested on MC3T3-E1 pre-osteoblast cells. MC3T3-E1 cells were cultured in α-MEM media containing 10% FBS (Invitrogen, Carlsbad, CA, USA), 2 mM L-glutamine, and antibiotics (100 units/mL penicillin and 0.1 mg/mL streptomycin, Invitrogen), as previously described [12,36]. Osteogenesis was induced by osteogenic media (OM) supplemented with 50 mg/mL ascorbic acid, 10 mM β-glycerophosphate (Sigma-Aldrich, St. Louis, MO, USA) and sodium alginate (500 µg/mL, Sigma-Aldrich). Cells were treated with CSA-90 (5 µM) or BBA-1 (5 µM) with or without rhBMP-2 (50 ng/mL). A p-nitrophenyl phosphate assay was performed to measure alkaline phosphatase activity (Sigma-Aldrich) and compared to untreated cells grown in OM. Assays were performed in triplicate with two independent repeats.

### 4.9. Protein Prenylation Assays

This assay was performed as previously described [19]. Cultured J774.2 macrophages were treated with ALN, CSA-90 or BBA-1 at a range of concentrations (10, 25, 50 µM). After 24 h of treatment, the cells were lysed by sonication in 50 mM HEPES, 2 mM MgCl2, 50 mM NaCl, 1 × Roche complete EDTA-free protease inhibitor cocktail. A total of 20 µg of protein was boiled with Laemmli sample buffer and separated via electrophoresis using 10–20% precast Criterion TGC gels (Bio-Rad, Hercules, CA, USA) and transferred onto PVDF-FL blots using a Trans-Blot Turbo (Bio-Rad). Blots were blocked with Odyssey Blocking Buffer (Li-COR) for 1 h at room temperature, washed with Tris-buffered saline, 0.1% Tween 20 (TBS-T) for 1 h, then incubated with 1/1000 dilution of primary antibodies that bind unprenylated Rap1A (Santa Cruz, sc1482) or prenylated Rap1A/B (Cell Signaling, 2399S) for 16 h at 4 °C, and LiCOR secondary antibodies (1/20,000 dilution anti-goat 680 RD, anti-rabbit 800 CW, respectively). Blots were washed 3 times with TBS-T and scanned using a Li-COR Odyssey^®^ CLx infrared imaging platform.

### 4.10. Statistics

Statistical analysis was performed using a one-way ANOVA with a post-hoc Tukey’s multiple comparison test (Graph Pad Prism 7). The cut-off for statistical significance was *p* < 0.05. Experimental measurements were performed in triplicate and the results are expressed as mean ± standard error measurement (SEM).

## 5. Conclusions

This paper describes a systematic approach for the creation of a novel bone binding antibiotic by combining a CSA with a bisphosphonate via a heterobifunctional PEG-based linker. The chemistry of the reaction and purity of the final compound was analyzed by HPLC elution using an evaporative light scattering detector, FT-IR, ^1^H-NMR and MS spectroscopy. Functional assays confirmed bone mineral affinity, pro-osteogenic activity, and antimicrobial activity, but a lack of effects on protein prenylation was seen with the parent bisphosphonate. These data support the further investigation of BBA-1 as an orthopedic antimicrobial, which would be supported by microbial infection prophylaxis in small and large animal models of osteomyelitis and implant-associated infection.

## 6. Patents

A.S., S.K., P.V., and P.B.S. hold intellectual property interests in bone binding antimicrobial compounds.

## Data Availability

The data presented in this study are available in article.

## Supplementary Materials

The following are available online, Figure S1: Mass spectrometry analysis spectrum of CSA-90. Distinctive doubly conjugated ions corresponding to CSA-90 was identified; Figure S2: Mass spectrometry analysis spectrum of ALN.

## Sample Availability

Not available.

## References

[1] Klenerman L. (2007). A history of osteomyelitis from the Journal of Bone and Joint Surgery. J. Bone Jt. Surg. Br..

[2] Conterno L.O., Turchi M.D. (2013). Antibiotics for treating chronic osteomyelitis in adults. Cochrane Database Syst. Rev..

[3] Mathews J.A., Ward J., Chapman T.W., Khan U.M., Kelly M.B. (2015). Single-stage orthoplastic reconstruction of Gustilo-Anderson Grade III open tibial fractures greatly reduces infection rates. Injury.

[4] Jorge L.S., Chueire A.G., Rossit A.R.B. (2010). Osteomyelitis: A current challenge. Braz. J. Infect. Dis..

[5] Malizos K.N. (2017). Global Forum: The Burden of Bone and Joint Infections: A Growing Demand for More Resources. J. Bone Jt. Surg. Am..

[6] Birt M.C., Anderson D.W., Bruce Toby E., Wang J. (2017). Osteomyelitis: Recent advances in pathophysiology and therapeutic strategies. J. Orthop..

[7] Waldvogel F.A., Papageorgiou P.S. (1980). Osteomyelitis: The past decade. N. Engl. J. Med..

[8] De Graeff J.J., Paulino Pereira N.R., van Wulfften Palthe O.D., Nelson S.B., Schwab J.H. (2017). Prognostic Factors for Failure of Antibiotic Treatment in Patients with Osteomyelitis of the Spine. Spine.

[9] Lai X.Z., Feng Y., Pollard J., Chin J.N., Rybak M.J., Bucki R., Epand R.F., Epand R.M., Savage P.B. (2008). Ceragenins: Cholic acid-based mimics of antimicrobial peptides. Acc. Chem. Res..

[10] Savage P.B., Li C., Taotafa U., Ding B., Guan Q. (2002). Antibacterial properties of cationic steroid antibiotics. FEMS Microbiol. Lett..

[11] Mills R., Cheng T.L., Mikulec K., Peacock L., Isaacs D., Genberg C., Savage P.B., Little D.G., Schindeler A. (2018). CSA-90 Promotes Bone Formation and Mitigates Methicillin-resistant *Staphylococcus aureus* Infection in a Rat Open Fracture Model. Clin. Orthop. Relat. Res..

[12] Schindeler A., Yu N.Y.C., Cheng T.L., Sullivan K., Mikulec K., Peacock L., Matthews R., Little D.G. (2015). Local Delivery of the Cationic Steroid Antibiotic CSA-90 Enables Osseous Union in a Rat Open Fracture Model of *Staphylococcus aureus* Infection. J. Bone Jt. Surg. Am..

[13] Reyes C., Hitz M., Prieto-Alhambra D., Abrahamsen B. (2016). Risks and Benefits of Bisphosphonate Therapies. J. Cell. Biochem..

[14] Young R.N., Grynpas M.D. (2018). Targeting therapeutics to bone by conjugation with bisphosphonates. Curr. Opin. Pharmacol..

[15] Russell R.G.G., Watts N.B., Ebetino F.H., Rogers M.J. (2008). Mechanisms of action of bisphosphonates: Similarities and differences and their potential influence on clinical efficacy. Osteoporos. Int..

[16] Roelofs A.J., Thompson K., Gordon S., Rogers M.J. (2006). Molecular mechanisms of action of bisphosphonates: Current status. Clin. Cancer Res..

[17] Farrell K.B., Karpeisky A., Thamm D.H., Zinnen S. (2018). Bisphosphonate conjugation for bone specific drug targeting. Bone Rep..

[18] Banerjee S.S., Aher N., Patil R., Khandare J. (2012). Poly(ethylene glycol)-Prodrug Conjugates: Concept, Design, and Applications. J. Drug Deliv..

[19] Ali N., Jurczyluk J., Shay G., Tnimov Z., Alexandrov K., Munoz M.A., Skinner O.P., Pavlos N.J., Rogers M.J. (2015). A highly sensitive prenylation assay reveals in vivo effects of bisphosphonate drug on the Rab prenylome of macrophages outside the skeleton. Small GTPases.

[20] Ye W.L., Zhao Y.-P., Li H.-Q., Na R., Li F., Mei Q.-B., Zhao M.-G., Zhou S.-Y. (2015). Doxorubicin-poly (ethylene glycol)-alendronate self-assembled micelles for targeted therapy of bone metastatic cancer. Sci. Rep..

[21] Hata K., Tokuhiro H., Nakatsuka K., Miki T., Nishizawa Y., Morii H., Miura M. (1996). Measurement of bone-specific alkaline phosphatase by an immunoselective enzyme assay method. Ann. Clin. Biochem..

[22] Dunford J.E., Thompson K., Coxon F.P., Luckman S.P., Hahn F.M., Poulter C.D., Ebetino F.H., Rogers M.J. (2001). Structure-activity relationships for inhibition of farnesyl diphosphate synthase in vitro and inhibition of bone resorption in vivo by nitrogen-containing bisphosphonates. J. Pharmacol. Exp. Ther..

[23] Epand R.F., Pollard J.E., Wright J.O., Savage P.B., Epand R.M. (2010). Depolarization, Bacterial Membrane Composition, and the Antimicrobial Action of Ceragenins. Antimicrob. Agents Chemother..

[24] Niemirowicz K., Surel U., Wilczewska A.Z., Mystkowska J., Piktel E., Gu X., Namiot Z., Akowska A.K.L., Savage P.B., Bucki R. (2015). Bactericidal activity and biocompatibility of ceragenin-coated magnetic nanoparticles. J. Nanobiotechnol..

[25] Gdowski A.S., Ranjan A., Sarker M.R., Vishwanatha J.K. (2017). Bone-targeted cabazitaxel nanoparticles for metastatic prostate cancer skeletal lesions and pain. Nanomedicine.

[26] Li B., Webster T.J. (2018). Bacteria antibiotic resistance: New challenges and opportunities for implant-associated orthopedic infections. J. Orthop. Res. Off. Publ. Orthop. Res. Soc..

[27] Konigsberg B.S., Valle C.J.D., Ting N.T., Qiu F., Sporer S.M. (2014). Acute Hematogenous Infection Following Total Hip and Knee Arthroplasty. J. Arthroplast..

[28] Sedghizadeh P.P., Sun S., Junka A.F., Richard E., Sadrerafi K., Mahabady S., Bakhshalian N., Tjokro N., Bartoszewicz M., Oleksy M. (2017). Design, Synthesis, and Antimicrobial Evaluation of a Novel Bone-Targeting Bisphosphonate-Ciprofloxacin Conjugate for the Treatment of Osteomyelitis Biofilms. J. Med. Chem..

[29] Tanaka K.S.E., Houghton T.J., Kang T., Dietrich E., Delorme D., Ferreira S.S., Caron L., Viens F., Arhin F.F., Sarmiento I. (2008). Bisphosphonated fluoroquinolone esters as osteotropic prodrugs for the prevention of osteomyelitis. Bioorg. Med. Chem..

[30] Lewis Phillips G.D., Li G., Dugger D.L., Crocker L.M., Parsons K.L., Mai E., Blattler W.A., Lambert J.M., Chari R.V.J., Lutz R.J. (2008). Targeting HER2-Positive Breast Cancer with Trastuzumab-DM1, an Antibody-Cytotoxic Drug Conjugate. Cancer Res..

[31] McCombs J.R., Owen S.C. (2015). Antibody Drug Conjugates: Design and Selection of Linker, Payload and Conjugation Chemistry. AAPS J..

[32] Ribeiro M., Monteiro F.J., Ferraz M.P. (2012). Infection of orthopedic implants with emphasis on bacterial adhesion process and techniques used in studying bacterial-material interactions. Biomatter.

[33] Houghton T.J., Tanaka K.S.E., Kang T., Dietrich E., Lafontaine Y., Delorme D., Ferreira S.S., Viens F., Arhin F.F., Sarmiento I. (2008). Linking Bisphosphonates to the Free Amino Groups in Fluoroquinolones: Preparation of Osteotropic Prodrugs for the Prevention of Osteomyelitis. J. Med. Chem..

[34] Kuroda K., Fukuda T., Okumura K., Yoneyama H., Isogai H., Savage P.B., Isogai E. (2013). Ceragenin CSA-13 induces cell cycle arrest and antiproliferative effects in wild-type and p53 null mutant HCT116 colon cancer cells. Anticancer Drugs.

[35] Van Bambeke F., Mingeot-Leclercq M.P., Struelens M.J., Tulkens P.M. (2008). The bacterial envelope as a target for novel anti-MRSA antibiotics. Trends Pharmacol. Sci..

[36] Schindeler A., Morse A., Peacock L., Mikulec K., Yu N.Y.C., Liu R., Kijumnuayporn S., McDonald M.M., Baldock P.A., Ruys A.J. (2010). Rapid cell culture and pre-clinical screening of a transforming growth factor-beta (TGF-beta) inhibitor for orthopaedics. BMC Musculoskelet. Disord..

