# Supplementary material for: Synthesis and Characterization of Bone Binding Antibiotic-1 (BBA-1), a Novel Antimicrobial for Orthopedic Applications
Source: Molecules. 2021 Mar 11;26(6):1541. doi: 10.3390/molecules26061541 (PMC7999004; doi:10.3390/molecules26061541)
Supplement: Supplementary file 1 [file molecules-26-01541-s001.pdf]

Supplementary Materials:

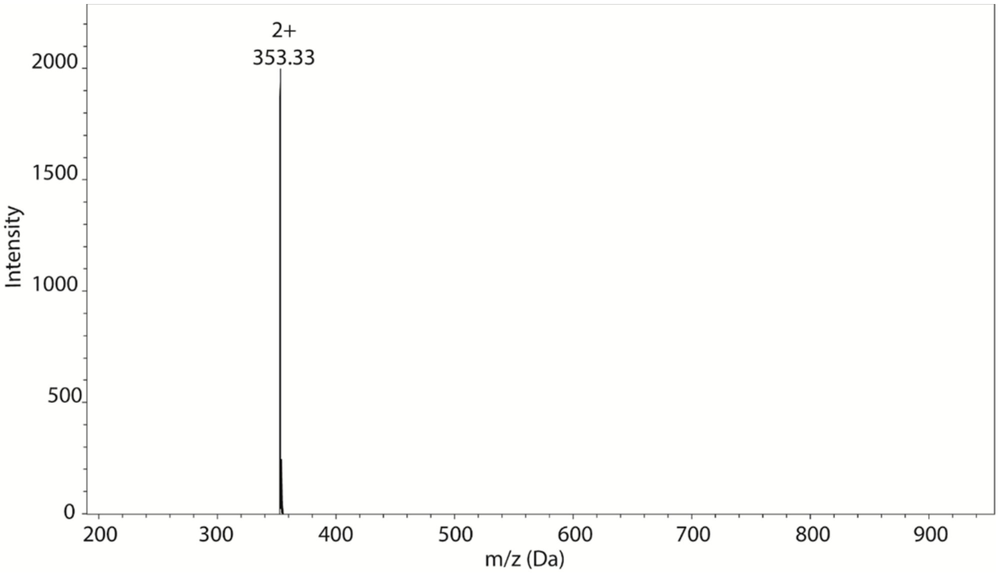

Figure S1: Mass spectrometry analysis spectrum of CSA-90. Distinctive doubly conjugated ions corresponding to CSA-90 was identified

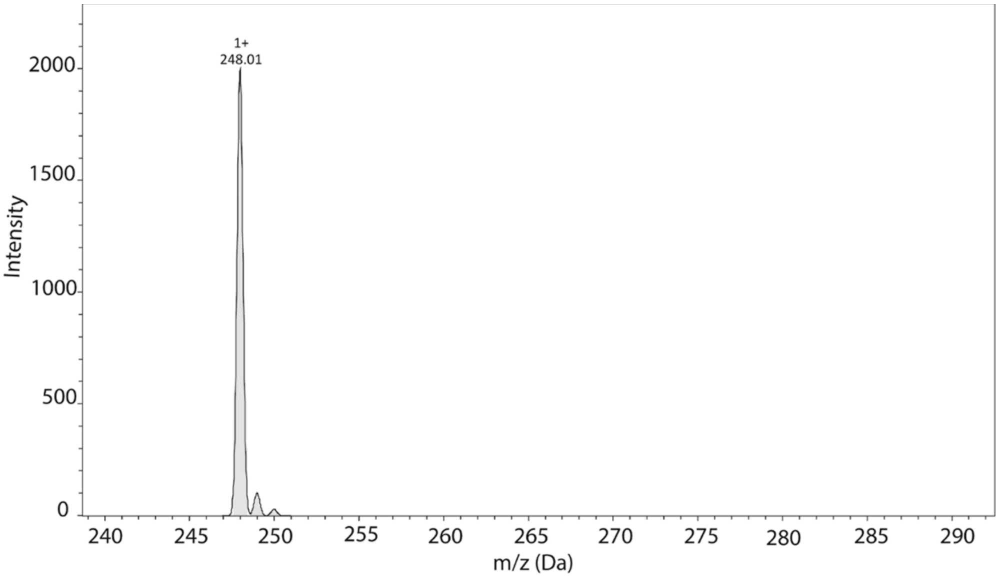

Figure S2: Mass spectrometry analysis spectrum of ALN
